# Research progress of hypoxia-inducible factor-1α and zinc in the mechanism of diabetic kidney disease

**DOI:** 10.3389/fphar.2025.1537749

**Published:** 2025-02-10

**Authors:** Wei Qin, Ping Nie, Xuejun Hui, Fei Chen, Xingbao Hu, Wenjiao Shi, Manyu Luo, Bing Li

**Affiliations:** Department of Nephropathy, The Second Hospital of Jilin University, Changchun, Jilin, China

**Keywords:** diabetic kidney disease, hypoxia inducible factor-1α, zinc, HIF-1α, diabetic nephropathy

## Abstract

Diabetic kidney disease is one of the common complications in diabetic patients and has gradually become an important pathogenic factor in chronic kidney disease. Therefore, studying the mechanisms of its occurrence and development is of great significance for the prevention and treatment of diabetic kidney disease. Some researchers have pointed out that there is a phenomenon of hypoxia in diabetic kidney tissue and believe that hypoxia-inducible factor-1α is closely related to the occurrence and progression of diabetic kidney disease. Additionally, the homeostasis of zinc plays a key role in the body’s adaptation to hypoxic environments. However, the specific relationship among these three factors remains unclear. This article provides a detailed review of the multiple roles of hypoxia-inducible factor-1α in the pathogenesis of diabetic kidney disease, including: regulating angiogenesis, increasing the expression of erythropoietin, modulating oxidative stress through the PI3K/AKT and HIF-1α/HO-1 pathways, promoting inflammatory cell infiltration and the release of inflammatory factors to induce inflammatory responses, facilitating epithelial-mesenchymal transition, pathological angiogenesis, and promoting the release of fibrotic factors, ultimately leading to renal fibrosis. Furthermore, HIF-1α also participates in the occurrence and development of diabetic kidney disease through mechanisms such as regulating apoptosis, inducing mitochondrial autophagy, and vascular calcification. At the same time, this article clarifies the regulatory role of the trace element zinc on hypoxia-inducible factor-1α in diabetic kidney disease. This article provides references and insights for further research on the pathogenesis and progression of diabetic kidney disease.

## Introduction

With improvements in economic level and lifestyle changes, the prevalence of diabetes is increasing. The global prevalence of diabetes in 2021 was estimated at around 536 million individuals, representing 10.5% of the total world population. Projections indicate that by the year 2045, this figure is expected to rise to 783 million, resulting in a prevalence rate of 12.2% (Sun et al., 2022). China currently holds the distinction of having the largest population of diabetes patients globally, representing approximately 25% of the worldwide diabetic demographic. As reported by the International Diabetes Federation, projections indicate that by the year 2045, the number of individuals diagnosed with diabetes in China may rise to 170 million, resulting in a prevalence rate of up to 12.5%. Notably, over 90% of diabetes cases in the country are classified as type 2 diabetes mellitus (T2DM). In individuals with diabetes, alterations in glucose metabolism elevate the metabolic demands associated with glucose, resulting in disruptions in renal hemodynamics as well as the influence of local hormones and cytokines within the kidneys. These elements collectively play a significant role in the development of diabetic kidney disease within the diabetic population. According to Fenta et al. , the global prevalence of chronic kidney disease (CKD) among patients with T2DM is 27% (Fenta et al., 2023). In China, the prevalence of DKD among adult patients with T2DM is reported to be 21.8% (Zhang et al., 2020). The rising prevalence of diabetes may be stabilizing, yet the increasing number of diabetes patients will continue to expand the future population of those afflicted by DKD. A national sampling survey reveals that only 40% of diabetes patients in China are cognizant of their condition, while two-thirds have not received any form of treatment. Furthermore, among those who are undergoing treatment, approximately 50% exhibit inadequate disease control (Wang et al., 2021). The considerable prevalence, limited awareness, and low rates of treatment success for diabetes in China, along with the gradual progression of the disease, its various complications, and its chronic characteristics, render diabetes a critical public health concern within the nation.

The etiology of diabetes is intricate and remains incompletely elucidated. Among the principal types of diabetes, T2DM is characterized by insufficient insulin secretion from pancreatic β-cells or by insulin resistance, resulting in elevated blood glucose levels. This, in turn, leads to an increase in harmful factors such as advanced glycation end products (AGEs), lipid metabolites, peroxides, inflammatory mediators, and iron. These damaging agents exert their influence on multiple target organs, including the kidneys, heart, and retina, prompting pathological alterations such as apoptosis in the cells of these organs, ultimately culminating in functional impairment of the affected tissues. DKD represents a significant microvascular complication associated with T2DM. Clinically, it is characterized by persistent albuminuria, a gradual deterioration in glomerular filtration rate, and in advanced cases, progression to end-stage renal disease (ESRD). The pathogenesis of DKD is also complex. It is currently posited that the development of DKD is attributable to a combination of hemodynamic alterations, metabolic disturbances, and their interactions, which culminate in oxidative stress and inflammatory responses within the renal system (Tuttle et al., 2022). Hypoxia is detectable in the renal medulla during the initial phases of disease advancement in DKD. Using oxygen microelectronics, Palm found that PO_2_ in the renal tissue of diabetic rats was much lower than that in normal mice (Palm et al., 2003). Ries et al. used blood oxygenation level-dependent magnetic resonance imaging (BOLD-MRI) to determine that deoxyhemoglobin signals increased in the outer medullary region of diabetic animals (Ries et al., 2003). Researchers observed through BOLD-MRI that 2 days post-induction of diabetes, the oxygen partial pressure in the kidneys of diabetic rats significantly decreased, with worsening hypoxia over time (Dos et al., 2007). Concurrently, in patients with DKD, a continuous decline in cortical PO_2_ was noted as the disease progressed (Yin et al., 2012). A study in China indicated that the prevalence of obstructive sleep apnea syndrome among hospitalized patients with T2DM could be as high as 60.1% (Zhang P et al., 2016). As a primary pathophysiological mechanism of obstructive sleep apnea syndrome, intermittent hypoxia may be common among diabetic patients. However, due to the current limitations of technologies such as BOLD-MRI in clinical applications, there is still relatively little data regarding hypoxia in patients with DKD. Therefore, there is an urgent need for larger sample size studies and longer follow-up research to further explore the hypoxic conditions in patients with DKD. Beyond the kidneys, diabetes can also induce hypoxia in other organs of the body, such as the retina (Arden and Sivaprasad, 2011), islets (Sato et al., 2011), adipose (Lee et al., 2014) and other tissues.

A substantial body of research has established that hypoxia is associated with multiple factors, including hemodynamic alterations (Damiani et al., 2021), metabolic dysfunctions (Wheaton and Chandel, 2011), oxidative stress and inflammatory responses (McGarry et al., 2018). This indicates that renal hypoxia serves as a fundamental pathogenic mechanism in the onset and advancement of DKD. The human body utilizes a variety of mechanisms to adapt to hypoxic conditions. In instances of acute hypoxia, sodium ions function as second messengers, influencing the processes of mitochondrial oxidative phosphorylation and altering the fluidity of the mitochondrial inner membrane, as well as the transmission of redox signals. These changes subsequently impact cellular metabolism, facilitating adaptation to the acute hypoxic state (Hernansanz-Agustín et al., 2020). In contrast, prolonged and chronic hypoxia is primarily regulated by hypoxia-inducible factor-1 (HIF-1). HIF-1α, the principal regulatory component for hypoxic adaptation, plays a significant role in the pathogenesis and progression of DKD through several mechanisms, including the induction of angiogenesis, upregulation of erythropoietin (EPO) expression, suppression of oxidative stress and inflammatory responses, regulation of apoptosis, promotion of mitochondrial autophagy, and inhibition of fibrosis and vascular calcification. Given its potential protective effects on the kidneys, HIF-1α has emerged as a focal point of research in recent years.

**FIGURE 1:**
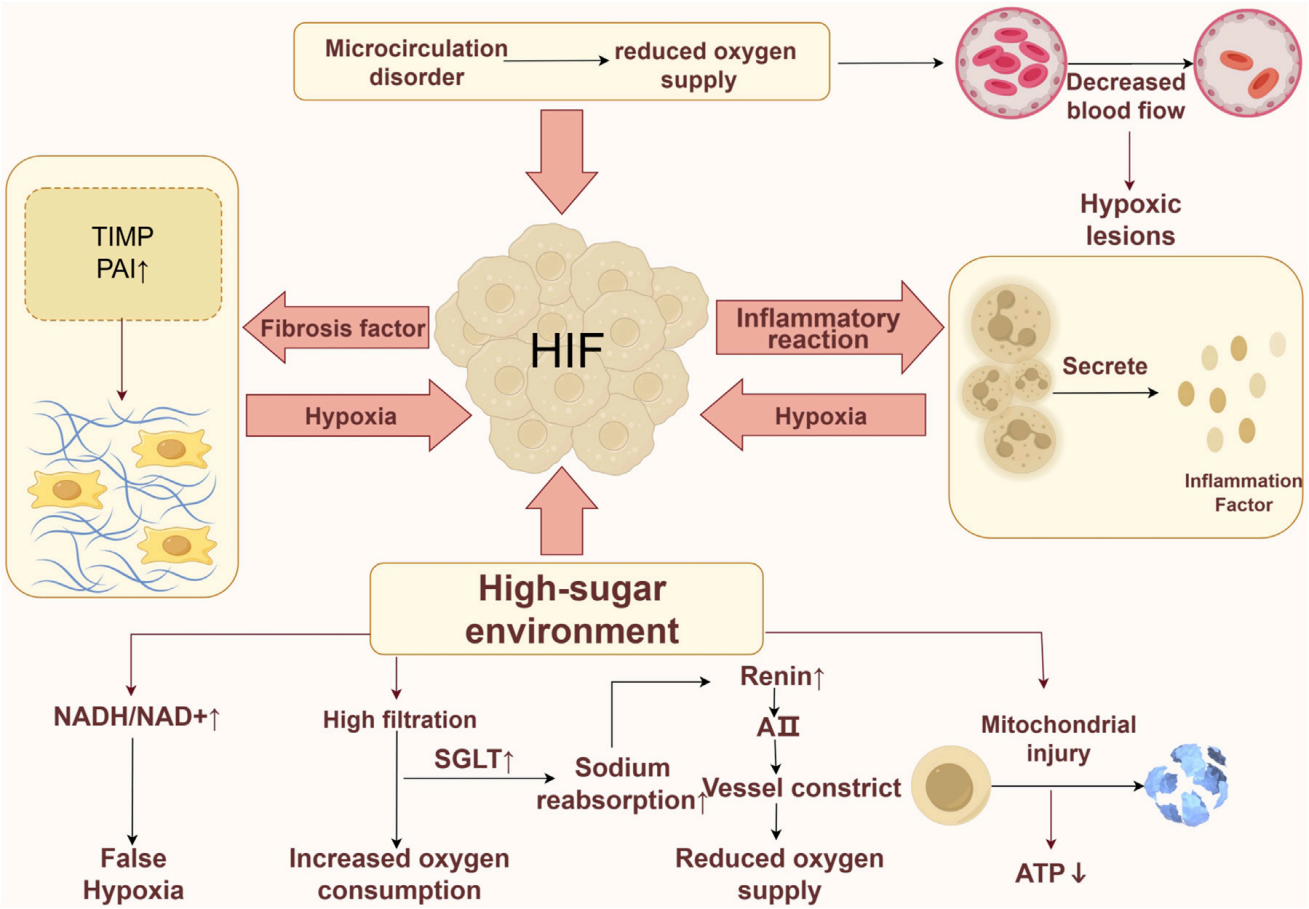
Hypoxia mechanism in DKD. The formation of a hypoxic environment can be induced by various factors. Firstly, in a high-glucose environment, the occurrence of pseudohypoxia is attributed to an imbalance in the NADH/NAD + ratio, while the excessive activation of SGLT leads to an increased consumption of oxygen and affects the release of Ang II, further impacting oxygen supply. Additionally, a high-glucose environment may impair mitochondria, resulting in reduced ATP production. Secondly, a vicious cycle is established among hypoxia, renal fibrosis, and inflammatory responses, exacerbating cellular hypoxia. Lastly, primary microcirculatory damage can lead to a decrease in microcirculatory blood flow, thereby reducing oxygen supply.

The hypoxic conditions observed in the kidneys may arise from multiple factors (Figure 1). In the context of elevated glucose levels, the metabolism of glucose via the polyol pathway is enhanced, leading to an increased ratio of reduced to oxidized nicotinamide adenine dinucleotide. This alteration contributes to a state referred to as “pseudohypoxia” within the organism (Williamson et al., 1993). Elevated sugar levels can trigger hyperfiltration in healthy glomeruli by enhancing the reabsorption of glucose and sodium in renal tubular epithelial cells via the sodium-glucose co-transporter (SGLT) located in the proximal tubule, which results in significant oxygen consumption. In individuals with DKD, ongoing inflammatory responses can release a substantial quantity of inflammatory factors, thereby elevating the basal metabolic rate and contributing to increased oxygen consumption within the body (Hansell et al., 2013). In addition to the increased consumption of oxygen, the reduction in oxygen supply is also a contributing factor to renal hypoxia. In the initial phases of DKD, impairment of the primary renal microcirculation can result in diminished blood flow and reduced oxygen delivery to the kidneys (Eckardt et al., 2005). Furthermore, the activation of SGLT enhances sodium reabsorption, which subsequently decreases the sodium concentration in the macula densa of the distal renal tubule. This decrease triggers an increase in the release of renin and angiotensin II (Ang II) (Yin et al., 2012; Zhu et al., 2011). The resultant constriction of the afferent arterioles by Ang II further compromises oxygen supply within the renal tubular interstitium (Hesp et al., 2020). Prolonged hypoxia in the kidneys of DKD patients can lead to damage in the renal tubular interstitium, causing an imbalance in the synthesis and degradation of the extracellular matrix (ECM), ultimately resulting in renal fibrosis. This fibrosis restricts oxygen transport and exacerbates the reduction in oxygen supply. Mitochondrial damage or dysfunction can exacerbate hypoxic injury. Mitochondrial dysfunction and reduced adenosine triphosphate (ATP) production can be observed even before the histological changes of diabetic kidney disease manifest (Imasawa et al., 2017). In essence, the development of a hypoxic microenvironment within renal tissue is primarily attributed to increased oxygen consumption coupled with decreased oxygen availability (Friederich-Persson et al., 2013). To adapt to hypoxic conditions, cells have evolved intricate mechanisms, with HIF-1α being among the first to respond to low oxygen levels, mediating the expression and regulation of a variety of downstream genes (Maxwell, 2003).

Numerous studies have demonstrated that trace elements may significantly influence the onset and progression of diseases associated with hypoxic conditions. Research suggests that the maintenance of zinc homeostasis is critical for the body’s ability to tolerate low oxygen environments (Guo et al., 2024b). Both animal and human subjects exposed to hypoxia have been observed to experience a reduction in plasma zinc levels (Guo et al., 2024a; Rawal et al., 1999; Vats et al., 2001), and appropriate zinc supplementation may mitigate symptoms related to hypoxia intolerance. Zinc is integral to the cellular regulation of HIF-1α. Evidence indicates that zinc can modulate the activity of HIF-1α through various mechanisms, underscoring the importance of intracellular zinc levels and homeostasis in preserving the normal function of HIF-1α (Nardinocchi et al., 2010). Consequently, zinc is pivotal in regulating the HIF-1α signaling pathway, which is crucial for cellular adaptation to hypoxic conditions. The pathogenesis of DKD is intricate, and the role of HIF-1α in mediating renal injury, along with its regulatory mechanisms, remains unclear. Further investigation is warranted to elucidate the regulatory effects of zinc on the HIF-1α signaling pathway in the context of DKD. This article reviews the research advancements surrounding HIF-1α and DKD, while exploring the clinical prospects of zinc as a therapeutic agent via the HIF-1α pathway for DKD, with the aim of providing reference and insight for future research.

## Structure and biological characteristics of HIF-1α

**FIGURE 2:**
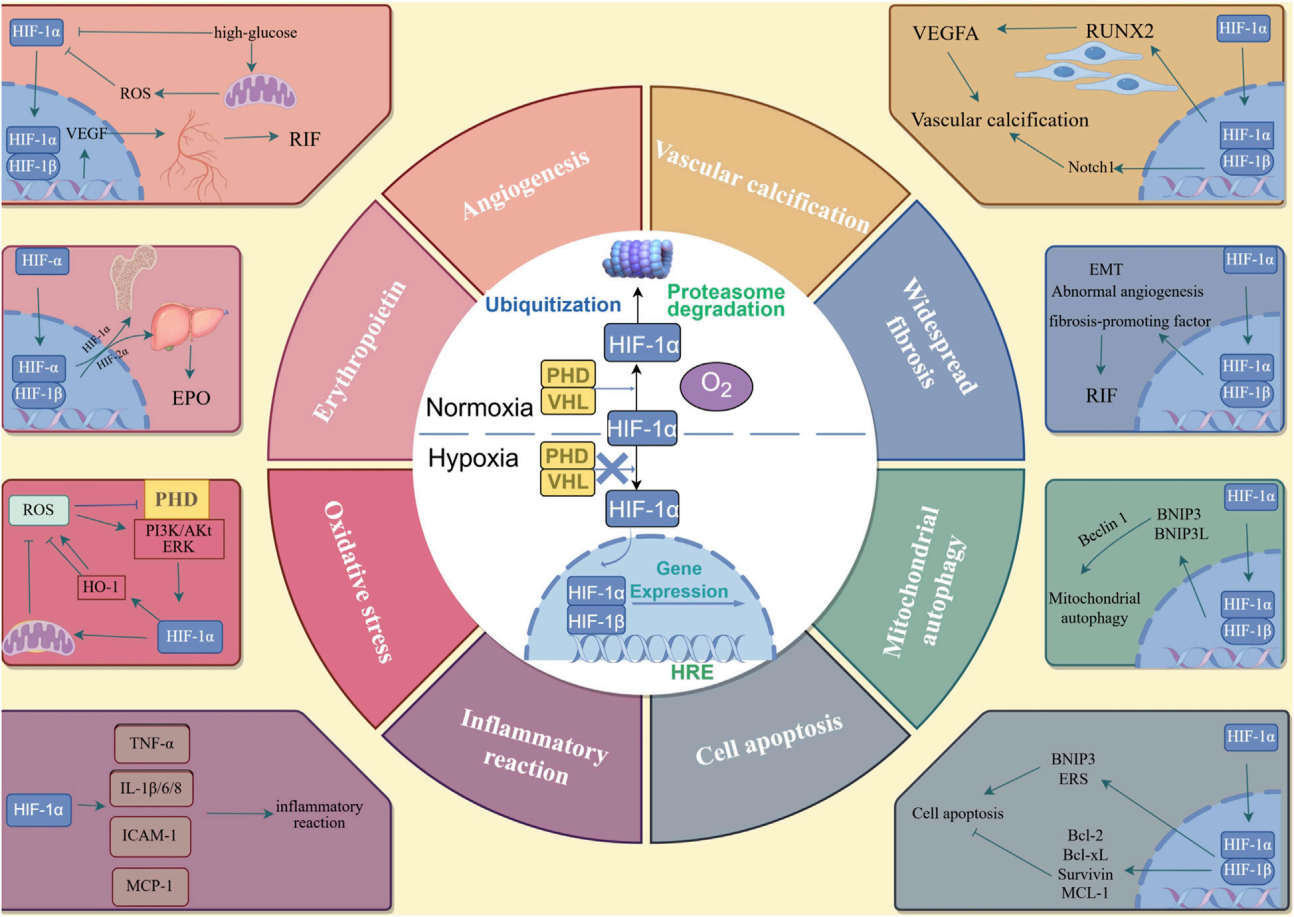
The metabolism of HIF-1α and the mechanism of HIF-1α in DKD. In the nomoxic conditions, proline residues on the HIF-1α protein are hydroxylated by PHD, and the hydroxylated HIF-1α is connected by VHL ubiquitin. HIF-1α is identified and ubiquitized, and finally hydrolyzed by protesome. In the hypoxia conditions, the activity of PHD is inhibited, and the degradation of the HIF-1α is blocked, HIF-1α is transferred to the nucleus and HIF-1β dimerized to form HIF-1. After combining with HRE, the transcription of multiple downstream target genes can be adjusted. The downstream genes can lead to angiogenesis; increased expression of EPO; oxidative stress; inflammatory reaction; cell apoptosis; mitochondrial autophagy; widespread fibrosis and vascular calcification.

HIF-1 mainly exists in the form of a heterogeneous dimer, consisting of HIF-1α subunit with sensitive oxygen concentration changes and HIF-1β subunit with stable expression (Maxwell, 2003). The primary physiological function of HIF-1 is regulated by HIF-1α. Under normal oxygen conditions, proline residues on the HIF-1α protein are hydroxylated by prolyl hydroxylase (PHD), and hydroxylated HIF-1α is connected by von Hippel-Lindau (VHL) ubiquitin. HIF-1α is identified, ubiquitinated, and finally hydrolyzed by the proteasome. Under normal oxygen conditions, HIF-1α could not be detected. In the hypoxic state, the activity of oxygen-dependent PHD is inhibited and the degradation of HIF-1α is blocked, resulting in the accumulation of HIF-1α. HIF-1α is transferred to the nucleus and HIF-1β dimerizes to form HIF-1. After combining with the hypoxia response element (HRE), the transcription of multiple downstream target genes can be regulated (Catrina and Zheng, 2021). Additionally, the expression of HIF-1α is not only affected by the hypoxic environment, but is also related to the regulation of insulin, insulin-like growth factors, AGEs, and blood sugar levels (Figure 2).

## The mechanism of HIF-1α in DKD

### Angiogenesis

Under hypoxic conditions, HIF-1α can increase the formation of new blood vessels in renal tissue cells (Peng et al., 2023). Studies have found that the content of vascular endothelial growth factor (VEGF) in the serum of diabetic patients is significantly higher than that in normal individuals, and is positively correlated with the content of serum HIF-1α (Katavetin et al., 2006).

The VEGF family and its receptors are important cellular signaling molecules that play a significant role in the regulation of neovascularization. As one of the downstream target genes of HIF-1α, hypoxia can promote nuclear aggregation of HIF-1α in human microvascular endothelial cells (Hahne et al., 2018).

In many instances, the adaptation to hypoxia is not achievable, and as the condition advances, the phenomena of capillary rarefaction and hypoxia in DKD continue unabated. This persistence may be attributed to the combined effects of hypoxia and elevated glucose levels on renal function in the context of DKD. The hyperglycemic environment can influence the transcription of VEGF in renal tissue cells by altering the stability and activity of HIF-1α (Cerychova and Pavlinkova, 2018). Under conditions of high glucose and hypoxia, there is an overactivation of reactive oxygen species (ROS) radicals, which leads to a reduction in VEGF expression and vascularization. This, in turn, triggers a compensatory response characterized by a decrease in hypoxia-induced angiogenesis. Concurrently, the high-glucose environment diminishes cellular responsiveness to hypoxia, resulting in decreased expression of HIF-1α target genes and a significant reduction in VEGF levels within the glomeruli of diabetic patients (Chou et al., 2002). This decline in VEGF expression contributes to podocyte apoptosis, which serves as an intermediary link between thrombotic microvascular disease and DKD (Hernández-Arteaga et al., 2018).

It is crucial to recognize that inappropriate activation of HIF-1α can have detrimental effects. Research conducted by Hakroush et al. demonstrated that the upregulation of VEGF expression in renal tubules led to capillary proliferation and an increase in fibroblast activity surrounding these tubules, which subsequently resulted in the accumulation of ECM and the development of renal fibrosis (Hakroush et al., 2009). Additionally, investigations involving VHL knockout transgenic mice revealed that elevated levels of HIF-1α and increased expression of the downstream target gene VEGF were associated with significant endothelial cell proliferation around the renal tubules, as well as heightened interstitial fibrosis within the renal tubules (Theilig et al., 2011). These findings indicate that excessive activation of HIF-1α may facilitate capillary proliferation in the vicinity of renal tubules and stimulate fibroblast proliferation through the overexpression of VEGF, leading to the accumulation of newly formed ECM in the interstitial spaces between renal tubules and capillary loops. This process ultimately results in the formation of fibrous tissue characterized by a dense network of capillaries surrounding cysts, contributing to the progression of renal fibrosis.

### Increased expression of EPO

EPO is a glycoprotein produced by endothelial cells in the kidneys and fibroblasts located in the peritubular interstitium. EPO interacts with EPO receptors on erythroid progenitor cells, facilitating their proliferation, differentiation, and maturation, while also enhancing the release of reticulocytes. The long-term hypoxic state of the kidneys in diabetic patients can induce the transformation of EPO-producing cells into α-smooth muscle actin (α-SMA) positive myofibroblasts, resulting in the loss of hypoxia-induced EPO expression. Patients suffering from DKD may experience anemia due to inadequate EPO production and a deficiency of hematopoietic components. In comparison to anemia associated with other forms of chronic kidney disease (CKD), the anemia linked to DKD manifests earlier, is more severe, and is associated with a poorer prognosis (Loutradis et al., 2016).

Research indicates that HIF-PHD inhibitors (HIF-PHI) can impede the hydroxylation of HIF-α by PHD, thereby diminishing the degradation of HIF-α. The resultant accumulation of HIF-α within cells facilitates the formation of the heterodimer HIF-1, which subsequently binds to HRE and stimulates the expression of downstream target genes, such as EPO and its receptor, thus enhancing erythropoiesis (Bernhardt et al., 2010). The HIF-β subunit is consistently expressed across various cell types, and its intracellular levels and biological activity remain unaffected by external hypoxic conditions, consequently, hypoxia primarily modulates EPO gene expression through HIF-α. There exist three isoforms of HIF-α: HIF-1α, HIF-2α, and HIF-3α (Holmquist-Mengelbier et al., 2006). Notably, HIF-1α is ubiquitously expressed in nearly all cell types, with a predominant presence in renal tubular cells. In contrast, HIF-2α is localized specifically to renal endothelial cells and fibroblasts adjacent to the renal tubules. Animal studies have revealed that the knockout of the HIF-2α gene in mice leads to a reduction in red blood cell count, hemoglobin levels, and hematocrit. Notably, PHD inhibitors did not exert any effects on mice deficient in HIF-2α (Gruber et al., 2007), indicating that HIF-2α serves as the principal regulator of EPO production under both physiological and stress-related conditions.

In comparison to HIF-2α, HIF-1α plays a more critical role in the preservation of hematopoietic stem cells within the hypoxic microenvironment of the bone marrow. Studies have demonstrated that hematopoietic stem cells cultured in conditions of extreme hypoxia can maintain the stable expression of HIF-1α (Takubo et al., 2010). Conversely, hematopoietic stem cells derived from HIF-1α deficient mice display notable functional impairments.

The above research indicates that HIF-1 can promote erythropoiesis by inducing the expression of endogenous EPO, thereby ameliorating renal anemia. Currently, HIF-PHI has entered the clinical research phase and is anticipated to become a novel therapeutic agent for the treatment of anemia.

### Oxidative stress

The activation of HIF-1α is intricately linked to oxidative stress. ROS play a crucial role in maintaining the stability of HIF-1α under normoxic conditions, as physiological concentrations of ROS serve as signaling molecules that modulate various physiological processes within the organism. In conditions of physiological hypoxia, the mitochondrial electron transport chain may be impaired due to inadequate oxygen availability, resulting in the excessive generation of ROS. Specifically, hydrogen peroxide, a component of ROS, can inhibit the enzymatic activity of PHD by activating signaling pathways mediated by small GTPases such as Rac or Rho. This inhibition leads to the stabilization of HIF-1α and the subsequent activation of transcription for its downstream target genes (Rabbani et al., 2010). Furthermore, ROS can prevent the degradation of HIF-1α by inhibiting PHD activity and activating the phosphatidylinositol 3-kinase/protein kinase B (PI3K/AKT) signaling pathway, as well as the extracellular signal-regulated kinase pathway, thereby elevating HIF-1 levels (Iacobini et al., 2022).

HIF-1 exhibits a negative feedback mechanism on the production of ROS. Under typical conditions, HIF-1 mitigates ROS generation through several pathways. Primarily, HIF-1 modifies energy metabolism in hypoxic environments via metabolic reprogramming, which subsequently diminishes ROS production from the mitochondrial electron transport chain. Additionally, HIF-1 promotes selective autophagy of mitochondria, facilitating the removal of damaged and senescent mitochondria, thereby further reducing ROS output. Moreover, HIF-1α can inhibit ROS production by obstructing the activity of the electron transport chain during mitochondrial respiration.

Additionally, HIF-1 regulates ROS production through the HIF-1/heme oxygenase-1 (HO-1) pathway. HO-1 is a stress-inducible enzyme that catalyzes the breakdown of heme into carbon monoxide, iron, and biliverdin. As a downstream target gene of HIF-1, the expression of HIF-1α in skeletal muscle and fibroblasts cells is elevated under hypoxic conditions, which enhances the transcription of HO-1 (Dunn et al., 2021) and provides cytoprotection by facilitating the clearance of ROS (Rosenberger et al., 2008). However, excessive heme degradation by HO-1 may lead to the accumulation of its degradation products within the cell, resulting in iron overload, which can exacerbate oxidative stress and lipid peroxidation, ultimately increasing ROS production (Feng et al., 2021). Additionally, HIF-1 can influence downstream target genes to enhance the transcription of nicotinamide adenine dinucleotide phosphate oxidase 4, thereby further promoting ROS generation (Diebold et al., 2010).

Chronic oxidative stress may influence the generation of HIF-1α through epigenetic modifications. Epigenetic regulation encompasses DNA methylation, histone modifications, and the modulation of non-coding RNAs. Research has shown that ROS can affect DNA methylation (Wu and Ni, 2015). Methylation of superoxide dismutase (SOD) can activate HIF-1α, thus participating in the regulation and function of the HIF-1 signaling pathway. Additionally, under hypoxic conditions, HIF-1α can also regulate epigenetic modifications (Choudhry and Harris, 2018). This suggests that epigenetic regulation may be a crucial mechanism in the modulation and function of HIF-1 in the hypoxic response associated with diabetes (Pang et al., 2021). Therefore, considering it as a potential therapeutic target merits further investigation.

### Inflammatory reaction

Under conditions of high-glucose and hypoxia, cells generate excessive ROS, which serve as a significant contributor to inflammation. Oxidative stress in glomerular mesangial cells stimulates the secretion of adhesion molecules, including intercellular cell adhesion molecule-1 and monocyte chemoattractant protein-1 (MCP-1), as well as inflammatory mediators such as nuclear factor-κB (NF-κB). Thioredoxin-interactingprotein (TXNIP) serves as a crucial regulatory factor in the activation of the nucleotide-binding oligomerization domain-like receptor protein 3 (NLRP3) inflammasome within the organism. It facilitates the dissociation of thioredoxin and TXNIP through the action of ROS, thereby enhancing the activation of the NLRP3 inflammasome by TXNIP. This process ultimately results in the production and release of interleukin-1β (IL-1β), which initiates an inflammatory response (Yang et al., 2020). While the inflammatory response functions as a protective mechanism against external stimuli, chronic inflammation can result in tissue damage. The heightened metabolic demands of numerous infiltrating inflammatory cells within renal tissue can induce hypoxia in the renal microenvironment, which in turn can activate endogenous inflammatory responses. This interaction establishes a detrimental cycle between hypoxia and inflammation, ultimately resulting in renal injury.

In individuals with DKD or in relevant animal models, there is a marked increase in the expression of pro-inflammatory genes, which correlates with elevated levels of inflammatory cytokines in peripheral blood (Liu et al., 2018). Histological examination reveals a substantial presence of lymphocytes, macrophages, and mast cells in the kidneys of patients with DKD, these immune cells are capable of secreting significant quantities of inflammatory mediators and cytokines that contribute to renal damage, either directly or indirectly (Navarro-González et al., 2011). Prior research has identified key inflammatory mediators, including tumor necrosis factor-α (TNF-α), interleukins 1 (IL-1), interleukin-6 (IL-6), and interleukin-18 (IL-18), MCP-1, NF-κB, and NLRP3 inflammasome, as critical players in the inflammatory processes associated with DKD (Wada and Makino, 2013; Wu et al., 2024; Zhu et al., 2024). This body of evidence underscores the significance of the inflammatory response in the pathogenesis and progression of DKD.

HIF-1α is implicated in the inflammatory response associated with DKD by facilitating the production of various cytokines. In hyperglycemic conditions, HIF-1α expression is upregulated, which enhances the inflammatory response in mesangial cells and markedly increases the levels of pro-inflammatory factors such as TNF-α, IL-1β, and IL-6, thereby exacerbating inflammatory damage to vascular endothelial cells. Zhao et al. demonstrated that human umbilical vein endothelial cells exposed to high glucose in a hypoxic environment for 48 h exhibited an 8.84-fold increase in HIF-1α mRNA expression compared to cells exposed solely to high glucose. Additionally, the expression of inflammatory factors IL-6, IL-8, intercellular cell adhesion molecule-1, and MCP-1 mRNA was significantly elevated, an effect that could be attenuated by the HIF-1 inhibitor KC7F2, indicating that HIF-1 exacerbates renal damage by promoting the expression of inflammatory factors during DKD (Zhao et al., 2021). Furthermore, the application of YC-1, a specific HIF-1α inhibitor, has been shown to enhance the inflammatory response in tubular epithelial cells and increase the production of cytokines IL-1β and IL-18 (Lee et al., 2018).

In summary, HIF-1α exacerbates vascular endothelial damage by promoting the infiltration of inflammatory cells and the release of inflammatory factors, creating a vicious cycle of hypoxia and inflammation in the kidneys that contributes to the progression of DKD (Table 1). Therefore, inducing the degradation of HIF-1α may emerge as an effective strategy to disrupt this vicious cycle, warranting exploration as a potential therapeutic target for DKD.

**TABLE 1:** The role of inflammatory factors in inflammation and their interactions with HIF-1.

Inflammatory factor	The role in inflammation	The interactions with HIF-1
TNF-α	Pro-inflammatory effects: TNF-α binds to TNF receptors on the cell surface, increasing the production of superoxide anions, stimulating cell degranulation and myeloperoxidase, which leads to the secretion of inflammatory factors such as IL-8 and IL-1 by endothelial cells, and promotes the adhesion of neutrophils to endothelial cells, thereby stimulating a local inflammatory response in the body. TNF-α can also stimulate monocytes and macrophages to produce and release IL-1 and IL-8, further exacerbating the inflammation Leukocyte mobilization: TNF-α promotes the mobilization and activation of leukocytes, enhancing the immune system’s response to infection or injury. It affects vascular endothelial cells, increasing vascular permeability and facilitating the entry of inflammatory cells Tissue damage: In cases of chronic inflammation or excessive activation, TNF-α can lead to tissue damage.	HIF-1 exerts an influence on cellular processes that enhances the expression of TNF-α, while TNF-α concurrently regulates the production of HIF-1. Specifically, TNF-α is capable of increasing both the activity of HIF-1 and the levels of HIF-1 DNA (Hellwig-Bürgel et al., 1999). Furthermore, TNF-α can upregulate the expression of HIF-1α through the NF-κB (Stiehl et al., 2002) and PI3K/Akt (Zhou et al., 2003) signaling pathways (Zhou et al., 2003)
IL-1β	induce the production of inflammatory mediators such as prostaglandins, nitric oxide, and cytokines, participating in the regulation of inflammatory responses, leading to symptoms such as vasodilation, increased permeability, and pain	The accumulation of HIF-1α can promote the expression of IL-1 (Zeng et al., 2024) and IL-1β can enhance the expression of HIF-1α mRNA (Thornton et al., 2000)
MCP-1	induce monocytes, microglia, and memory T lymphocytes possess the ability to migrate and infiltrate areas of injury and infection across a range of pathological conditions (Shimobayashi et al., 2018)	HIF-1α promotes the secretion of MCP-1, enhances the recruitment of monocytes and macrophages, and increases inflammation and fibrosis (Li et al., 2016)
NLRP3	The NLRP3 inflammasome activates the inflammatory response by maturing and secreting pro-inflammatory cytokines IL-1β and IL-18 (Broz and Dixit, 2016)	HIF-1 can interact with NLRP3 and participate in the inflammatory response by regulating NF-κB, promoting the activation and secretion of IL-1β. (Machado et al., 2022)
NF-κB	Regulate the expression of various genes related to interleukin, apoptosis inhibitors, and adhesion factor coding genes	TLR4 has the capacity to activate HIF-1α via the NF-κB signaling pathway, while HIF-1α can also directly modulate the TLR4-NF-κB signaling cascade. (Zhao et al., 2022) Hypoxia induces the expression of HIF-1α, which subsequently participates in the regulation of NLRP3 inflammasome pathway activation through NF-κB signaling. (Wang et al., 2024)

### Widespread fibrosis

A prolonged inflammatory response induces the infiltration of inflammatory cells into the kidneys, which in turn activates interstitial kidney cells, prompting them to release inflammatory molecules. This cascade incites vascular remodeling, ECM deposition, and mesangial proliferation, all of which contribute to the progression of renal fibrosis. Renal interstitial fibrosis (RIF) represents the primary pathological foundation and the ultimate common pathway for the progression of CKD to ESRD. This condition is characterized by the activation of fibroblasts and a substantial accumulation of ECM, which detrimentally impacts both the glomeruli and the overall kidney structure. Notably, the tubular basement membrane undergoes thickening, and there is an increase in the mesangial matrix, ultimately resulting in glomerulosclerosis and RIF. Research utilizing animal models has demonstrated that the expression of HIF-1α is elevated in the kidneys and ECM of diabetic model rats (Tang et al., 2012). In the context of DKD, hypoxic conditions can enhance the expression of HIF-1α. The sustained expression of HIF-1α may lead to glomerular hypertrophy and an increase in ECM, thereby facilitating the fibrotic process, generating significant scar tissue, disrupting the original tissue architecture of the kidneys, diminishing blood supply, and ultimately resulting in irreversible kidney dysfunction. In severe instances, this may culminate in renal failure.

**FIGURE 3:**
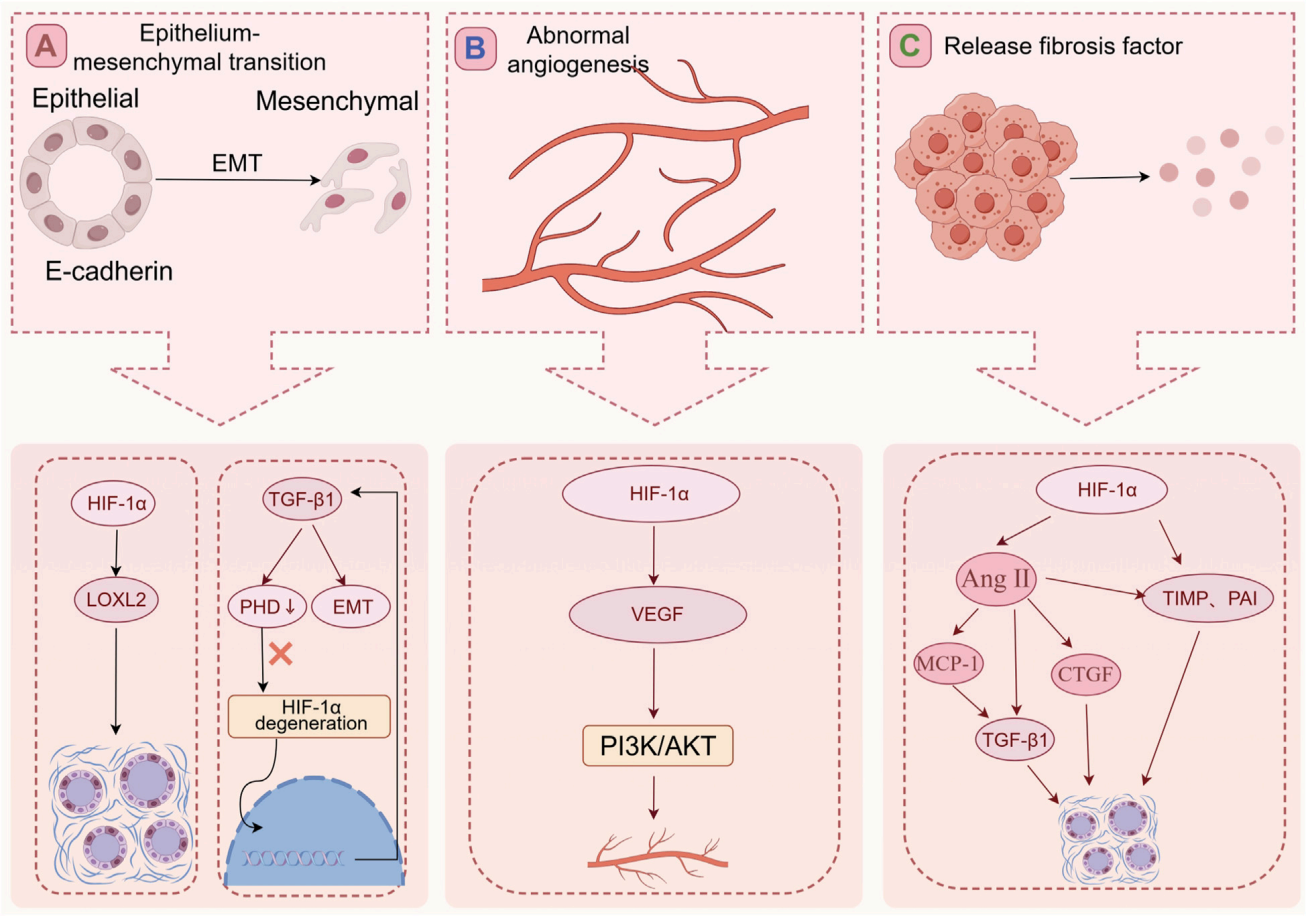
The mechanism of RIF. **(A)** HIF-1α can increase the expression LOXL2 to promote fibrosis. The TGF-β1 reduces the expression of PHD2 resulting in HIF-1α accumulation, and this PHD2/HIF-1α signaling pathway mediates TGF-β1-induced EMT in renal tubular cells. **(B)** The HIF-1α/VEGF signaling pathway of glomerular endothelial cells is activated, and the increased expression of VEGF can promote activation of signal path PI3K/AKT. The activation of the signal pathway leading to abnormal angiogenesis, exacerbating glomerular fibrosis, and ultimately the loss of glomerular function. **(C)** In a high-glucose and hypoxia environment, HIF-1α expression increases, leading to the activation of fibrosis-promoting factors such as TIMP and PAI. HIF-1α can also activate Ang II. Ang II further stimulates TGF-β1 expression and promotes pro-fibrotic factors, including CTGF, contributing to RIF by increasing ECM synthesis.

The potential mechanisms through which HIF-1α contributes to RIF are as follows:

Epithelium-mesenchymal transition (Figure 3A).

Epithelial-mesenchymal transition (EMT) is a biological process involving the dedifferentiation of epithelial cells, which is marked by the loss of cell polarity and specific epithelial markers, reorganization of the cytoskeleton, and the acquisition of a mesenchymal phenotype. This transformation enables epithelial cells to become fibroblast-like cells that are capable of secreting ECM components, which may accumulate in damaged renal tissues. A hyperglycemic and hypoxic environment can directly induce EMT in renal tubular epithelial cells and simultaneously enhance the expression of HIF-1α. The reduction of HIF-1α levels may mitigate EMT, indicating that HIF-1α is a critical factor in the EMT associated with DKD (Yang et al., 2021).

Research has shown that the activation of HIF-1α can enhance the expression of lysyl oxidase-like 2 (LOXL2), thereby facilitating the transition of epithelial cells to an interstitial phenotype, a process that contributes to fibrosis (Higgins et al., 2007). LOXL2, a member of the lysine oxidase family, is integral to the covalent crosslinking of collagen and elastin in the ECM, which is essential for the induction of EMT. Research has demonstrated that Rho-kinase inhibitors facilitate the degradation of HIF-1, inhibit the dimerization of HIF-1α with HIF-1β, and suppress the expression of their target genes (Matoba et al., 2013). Consequently, this leads to a decrease in the accumulation of glomerular mesangial matrix in diabetic patients and alleviates glomerular fibrosis. Silent information regulator 2 (Sirt1) has been shown to inhibit the activation of HIF-1α, resulting in a reduction of HIF-1α expression and a subsequent decrease in inflammation and fibrosis of glomerular cells under hyperglycemic conditions (Huang et al., 2015). Additionally, YC-1, a selective inhibitor of HIF-1α, has been found to significantly improve renal function and mitigate pathological injury in diabetic mice, as well as reduce the severity of renal fibrosis. Researchers have observed that the transfection of renal tubular cells with HIF-1α siRNA significantly inhibits transforming growth factor-β1 (TGF-β1) induced EMT. Furthermore, they have demonstrated that TGF-β1 can reduce PHD2 expression through a Smad-dependent mechanism, resulting in the accumulation of HIF-1α and consequently inducing EMT in renal tubular cells (Han et al., 2013).

The aforementioned study reveals that in DKD, HIF-1α induces EMT through pathways that promote the upregulation of LOXL2 and TGF-β1, leading to increased ECM generation and fostering RIF.

Abnormal angiogenesis (Figure 3B).

In DKD as we described above, abnormal neovascularization predominantly involves the formation of immature blood vessels, which can lead to renal fibrosis and, ultimately, a decline in glomerular function. The VEGF family and its associated receptors serve as critical signaling molecules that govern the process of neovascularization. Under conditions of elevated glucose, the HIF-1α/VEGF signaling pathway is activated in glomerular endothelial cells, resulting in an upregulation of VEGF expression. This increase in VEGF can subsequently activate the phosphorylation of endothelial nitric oxide synthase, which in turn leads to abnormal angiogenesis, exacerbates glomerular fibrosis, and ultimately contributes to the deterioration of glomerular function (Mahdy et al., 2010).

Activate the fibrosis-promoting factor (Figure 3C).

In environments characterized by elevated glucose levels and hypoxia, there is an upregulation of HIF-1α expression. HIF-1α has the capacity to activate downstream fibrosis-promoting factors, including tissue inhibitors of metalloproteinase (TIMP) and plasminogen activator inhibitor (PAI), which subsequently stimulate the synthesis of interstitial collagen and inhibit the degradation of the ECM. Furthermore, Ang II has the capacity to directly stimulate the expression of TGF-β1 molecules, which subsequently enhances TGF-β1 expression through the upregulation of cytokines such as MCP-1 (Zhou et al., 2006). This mechanism facilitates the accumulation of interstitial collagen, ultimately resulting in RIF. Furthermore, Ang II can elevate the expression of connective tissue growth factor (CTGF), a key mediator of fibrosis, through both direct and indirect pathways (Hussain et al., 2008). Additionally, Ang II may induce the expression of other pro-fibrotic factors, including a significant promotion of PAI transcription (Doller et al., 2009) and the activation of TIMP. These actions contribute to an increase in ECM synthesis while simultaneously decreasing its degradation, thereby culminating in the development of RIF. Additionally, both *in vivo* and *in vitro* studies have demonstrated that HIF-1α can also activate Ang II, thereby facilitating renal interstitial fibrosis (Tang et al., 2012). Krüppel-like factor 5 (KLF5) is expressed in the proximal tubular cells of fibrotic kidneys and may promote the expression of TGF-β1 through the transcriptional regulation of HIF-1α, thereby contributing to the onset and progression of kidney fibrosis. Studies indicate that, elevated concentrations of MK-8617, a HIF-PHI, have the potential to exacerbate renal tubular interstitial fibrosis through the activation of the HIF-1α/KLF5/TGF-β1 signaling pathway (Li et al., 2019).

In conclusion, HIF-1α exacerbates the RIF process in patients with DKD by inducing EMT, promoting pathological angiogenesis, and elevating the expression of profibrotic factors.

### Cell apoptosis

HIF-1α exhibits a dual role in regulating apoptosis, functioning both to promote and inhibit this process. HIF-1α can activate the gene encoding induced nitric oxide synthase, which facilitates the induction of apoptosis in cells. Furthermore, HIF-1α interacts with HRE to enhance the expression of B-cell lymphoma-2 (Bcl-2) interacting protein 3 (BNIP3), a member of the Bcl-2 family, which is also implicated in the promotion of apoptosis. Additionally, endoplasmic reticulum stress (ERS) represents a significant pathogenic mechanism contributing to the onset and progression of DKD. Moderate ERS contributes to the restoration of cellular homeostasis. However, excessive activation of ERS results in apoptosis. In cardiomyocytes, HIF-1α has the capacity to activate ERS, thereby facilitating intermittent hypoxia and triggering apoptotic processes (Moulin et al., 2022). This intervention enhances the ERS response in diabetic mice, thereby mitigating renal pathological damage and delaying the deterioration of renal function. The mechanism underlying HIF-1α-induced ERS may involve the activation of ERS through its transcriptional activity, which promotes the expression of genes associated with apoptosis (Sun et al., 2020). It has also been shown that HIF-1α can directly participate in the protein kinase R-like endoplasmic reticulum kinase (PERK) pathway (Moulin et al., 2020).

As a significant regulator of apoptosis that operates downstream of HIF-1α, the anti-apoptotic protein Bcl-2 is subject to direct transcriptional regulation by HIF-1α. This includes members of the Bcl-2 family, such as B-cell lymphoma-extra large (Bcl-xL), as well as myeloid cell leukemia-1 and Survivin, which belongs to the inhibitor of apoptosis protein family (Chen et al., 2009). Dexmedetomidine can increase the level of HIF-1α after renal ischemia-reperfusion injury in diabetic rats, activate the anti-apoptotic protein Bcl-2, and inhibit the apoptotic protein, thus improving cell vitality, reducing apoptosis and protecting kidney function (Ji et al., 2019).

### Mitochondrial autophagy

Mitochondria engage in selective self-removal through the process of autophagy to regulate their content and ensure quality control, thereby preserving mitochondrial homeostasis. A reduction in mitochondrial autophagy levels has been documented in mouse renal tubular epithelial cells subjected to high glucose conditions, as well as in kidney biopsy specimens from patients diagnosed with DKD (Zhan et al., 2015). This observation underscores the significant role of mitochondrial autophagy in the pathogenesis of DKD.

In experimental settings, researchers employed cobalt chloride to create a hypoxic environment in renal tubular epithelial cells. The application of a HIF-1α inhibitor resulted in decreased expression of HIF-1α and exacerbated mitochondrial damage within these cells (Jiang et al., 2020), suggesting that HIF-1α may confer protective effects on mitochondria under hypoxic conditions. Notably, hypoxia is associated with an increase in HIF-1α expression, which in turn enhances the expression of autophagy-related genes, including LC3, Atg5, Beclin 1, Atg7, and Bcl-2/BNIP3 (Chen et al., 2018). This indicates that HIF-1α may play a crucial role in maintaining mitochondrial homeostasis by promoting mitochondrial autophagy.

Chronic moderate hypoxia has been shown to elevate HIF-1α levels, which subsequently dimerizes with HIF-1β to form the HIF-1 complex. This complex enhances the expression of downstream target genes associated with cell death, such as BNIP3 and its homolog BNIP3L, by directly binding to the HRE located in their promoter regions, thereby inducing mitochondrial autophagy (Li et al., 2022). Furthermore, following the upregulation of BNIP3 expression by HIF-1α, there is also an increase in Beclin 1 levels, which occurs through the disruption of the interaction between Beclin 1 and Bcl-2, further promoting mitochondrial autophagy (Zhang et al., 2008). Under hypoxic conditions, HIF-1α facilitates mitochondrial autophagy by enhancing the expression of its downstream target genes, BNIP3 and BNIP3L, thereby sustaining mitochondrial homeostasis, improving mitochondrial morphology and function, and ultimately providing protection to renal tissues. Additionally, HIF-1α has the potential to upregulate genes encoding proteins involved in the mitochondrial tricarboxylic acid cycle, as well as proteins regulated by autophagy, thereby contributing to enhancements in mitochondrial morphology and functionality (Thomas et al., 2017).

### Vascular calcification

Vascular calcification represents a pathological phenomenon akin to advanced arteriosclerosis characterized by ossification. This process affects the endothelium or media and is correlated with heightened cardiovascular morbidity and mortality among individuals with diabetes or ESRD (Dhakshinamoorthy et al., 2017). Vascular calcification is an active, complex, and chronic process characterized by inflammation, oxidative stress, and apoptosis. Vascular smooth muscle cells (VSMCs) are the primary cell types in the vascular interstitium, contributing to arterial medial calcification through the phenotypic conversion of osteotropters (McCarty and DiNicolantonio, 2014). Prolonged hypoxia in the kidneys induces an upregulation of HIF-1α expression in renal tubular and interstitial cells. This increase in HIF-1α levels may directly or indirectly interfere with mineral metabolism in the bloodstream, ultimately contributing to the development of vascular calcification.

Extensive research has established that HIF-1α is integral to the process of vascular calcification. An analysis of computed tomography scan data from 405 patients diagnosed with type 2 diabetes demonstrated a significant increase in serum HIF-1α concentrations correlating with the advancement of coronary calcification, as evidenced by a positive relationship with the coronary artery calcification score. Furthermore, serum HIF-1α levels were found to be independently associated with both the presence and severity of vascular calcification. *In vitro* studies utilizing human aortic smooth muscle cells cultured under hypoxic conditions revealed a time-dependent induction of ECM calcification mediated by HIF-1α (Zhu et al., 2018). Hypoxic conditions were shown to synergistically enhance vascular calcification in conjunction with inorganic phosphates, primarily through increased expression of HIF-1α (Mokas et al., 2016). These findings highlight the critical role of HIF-1α in the mechanisms underlying vascular calcification.

In CKD rats, there was a notable increase in nuclear staining for HIF-1α. Furthermore, two critical regulatory genes downstream of HIF-1α, namely, vascular endothelial growth factor A and Notch1 protein, also exhibited significant upregulation (Mokas et al., 2016). The inhibition of HIF-1α resulted in a decrease in the levels of vascular endothelial growth factor A and Notch1 proteins, indicating that these proteins may act as downstream mediators of vascular calcification associated with HIF-1α. Additionally, runt-related transcription factor 2 (RUNX2) is identified as a pivotal factor in the reprogramming of VSMCs during vascular calcification. Under both normoxic and hypoxic conditions, the expression of RUNX2 was markedly diminished in cells following the specific knockout of HIF-1α in VSMCs (Guo et al., 2024). This observation suggests that HIF-1α may regulate vascular calcification by enhancing RUNX2 expression in VSMCs, with RUNX2 also interacting with HIF-1α in osteoblasts to modulate its stability and transcriptional activity. Collectively, these findings indicate that HIF-1α can facilitate the osteogenic transformation and calcification of VSMCs, thereby contributing to the vascular calcification process in CKD. Moreover, the activation of HIF-1α may serve as an independent predictor of vascular calcification.

In the early stages of DKD, HIF-1α facilitates the adaptation of renal surrounding tissues and cells to hypoxic environments by regulating the expression of downstream target genes and promoting neovascularization, thereby alleviating ischemia and hypoxia’s detrimental effects on the kidneys and delaying disease progression. However, as DKD progressively worsens, prolonged hyperglycemia suppresses the expression of HIF-1α, leading to diminished protective effects. Additionally, an increasingly pronounced phenomenon of HIF-1α overinduction promoting renal cell apoptosis emerges, further fostering fibrosis, commencing the manifestations of renal fibrosis, exacerbating vascular calcification, and ultimately inflicting severe damage on the structure and function of the kidneys, thereby accelerating disease advancement.

Given the multifaceted roles of HIF-1α in DKD, developing targeted therapeutic strategies or drugs aimed at HIF-1α may represent a promising direction for future treatments of DKD. Currently, a growing number of researchers have commenced in-depth investigations into HIF-1α and its downstream pathways to identify effective approaches for treating DKD.

Li et al. conducted a study demonstrating that the administration of miRNA-122-5p analogs via tail vein injection in rat models of streptozotocin-induced DKD resulted in a notable decrease in renal tubular injury and interstitial fibrosis. This finding suggests that miRNA-122-5p may confer a protective effect on renal tissues in hyperglycemic conditions. *In vitro* investigations revealed that the transfection of cells exposed to high glucose levels with miRNA-122-5p led to a significant downregulation of factor-inhibiting HIF-1 (FIH-1) expression. Furthermore, in a hyperglycemic environment, the overexpression of FIH-1α was associated with increased cell apoptosis, whereas the introduction of miRNA-122-5p markedly reduced apoptotic rates. Additionally, miRNA-155-5p was found to enhance HIF-1α activity by inhibiting FIH-1 expression, thereby potentially delaying the progression of DKD (Cheng et al., 2022). Liu et al. explored the induction of kidney fibrosis in diabetic mice through intraperitoneal albumin injection, coupled with oral administration of the pyruvate kinase M2 activator TEPP-46. Their findings indicated that TEPP-46 mitigated HIF-1α accumulation and exerted anti-fibrotic effects by activating pyruvate kinase M2 and enhancing pyruvate kinase activity (Liu et al., 2021). Angelamellisy Revelian Ndibalema and colleagues reported that empagliflozin could alleviate damage to renal tubular epithelial cells in high-glucose environments by promoting HIF-1α production (Ndibalema et al., 2020). Pang et al. observed that administering hirudin to DKD mice over a 16-week period inhibited the HIF-1α/VEGF signaling pathway, resulting in reduced ECM deposition and improved renal fibrosis in the DKD model (Pang et al., 2020). Moreover, the HIF-PHI roxadustat was shown to prevent the hydroxylation of HIF-1α by PHD domain enzymes, leading to HIF-1α accumulation in the cytoplasm, followed by its translocation to the nucleus where it forms heterodimers with HIF-1β. This process promotes the expression of downstream target genes such as EPO, thereby ameliorating renal anemia. Current research on roxadustat has progressed to phase III clinical trials in various countries, indicating its efficacy in improving anemia and other effects of CKD patients. Other investigations have demonstrated that another HIF-PHI, Enarodustat, can inhibit the metabolic shift from the tricarboxylic acid cycle to glycolysis, thereby enhancing energy metabolism in the early stages of DKD (Hasegawa et al., 2020).

The aforementioned mechanism highlights the significant role of HIF-1α in the pathogenesis and progression of DKD (Figure 2), positioning HIF-1α as a promising target for therapeutic intervention in this condition. Recent investigations have identified various strategies and pharmacological agents capable of inhibiting HIF-1α activity and its associated pathways. The mechanisms of action of these agents predominantly involve multiple facets of the HIF-1α activation pathway, encompassing transcription, translation, stability, nuclear transport, heterodimerization, and the regulation of downstream target gene expression. Notably, anthracycline drugs and amifostine have been shown to suppress the expression of HIF-1α mRNA, while the active component of AFP-464, amino flavone, can completely inhibit the expression of HIF-1α protein. Furthermore, EZN-2968, mTOR inhibitors (rapamycin), COX-2 inhibitors (ibuprofen), topoisomerase inhibitors (mitoxantrone), and cardiac glycosides have all demonstrated the ability to obstruct the translation of HIF-1α mRNA (Qannita et al., 2024). Currently, research efforts aimed at inhibiting HIF-1α activity and its related pathways are predominantly concentrated in the oncology domain, with a notable deficiency in studies pertaining to renal diseases, particularly DKD.

Current research on HIF-1α in the context of renal diseases predominantly addresses the management of renal anemia, a topic that has been extensively reviewed in prior literature. Recent investigations have demonstrated that the administration of dapagliflozin in animal models of progressive DKD significantly ameliorates hyperglycemia and reduces urinary albumin excretion. Longitudinal studies indicate that this pharmacological agent can suppress the expression of HIF-1 in the nuclei of both glomerular and tubular cells, leading to improvements in glomerulosclerosis and RIF (Inada et al., 2022). Furthermore, the application of the HIF-1α inhibitor PX-478 in streptozotocin-induced diabetic murine models has yielded promising results, including the restoration of normal insulin secretion in response to glucose from the pancreatic islets, a reduction in blood glucose levels, and an enhancement of β-cell functionality. *In vitro* studies utilizing human pancreatic organoids have also shown that PX-478 can augment glucose-stimulated insulin secretion. These findings suggest that HIF-1α inhibitors may play a role in mitigating β-cell exhaustion by enhancing the activity of pancreatic β-cells under conditions of metabolic stress. Nonetheless, it is important to note that this research has primarily focused on the effects of HIF-1α inhibitors in diabetic animal models and human islets *in vitro*; thus, further investigation is necessary to ascertain their effects on pancreatic β-cells within the human physiological context. These inhibitors hold potential as effective therapeutic agents for the long-term management of diabetes in the future (Ilegems et al., 2022).

There are several critical areas within HIF-1α research that necessitate further investigation. Firstly, while HIF-PHI has demonstrated some effectiveness in the management of renal anemia, its inherent shortcomings and limitations warrant careful consideration. Given the variability in the distribution and function of HIF subtypes across renal cells, prolonged administration of HIF-PHI may result in the accumulation of HIF-1α, which could lead to adverse outcomes such as renal fibrosis, pathological angiogenesis, vascular calcification, and the promotion of tumorigenesis. Consequently, it is imperative to conduct clinical trials targeting specific patient populations to elucidate its therapeutic efficacy. Secondly, the mechanisms by which HIF-1α contributes to the onset and progression of DKD remain inadequately understood. In particular, there is a pressing need for in-depth exploration of the direct effects of various drug classes, including HIF-PHD inhibitors and SGLT-2 inhibitors, on HIF-1α signaling pathways.

## Regulation of HIF-1α in DKD with zinc

Research has substantiated the regulatory function of trace elements on HIF-1α within the organism. Specifically, studies indicate that manganese ions are capable of downregulating PHD2, thereby activating the protein degradation pathway associated with HIF-1α, which results in a reduction of HIF-1α levels (Chen et al., 2024). Furthermore, iron is also critical in modulating HIF-1α expression, it not only facilitates the translation of HIF-1α transcripts but also activates PHD, which catalyzes the VHL-dependent degradation of HIF-1α (Linehan et al., 2019). Similarly, elevated concentrations of copper ions can inhibit the stabilizing effect of PHD on HIF-1α, thereby enhancing the transcriptional activity of HIF-1. In addition to the aforementioned trace elements, zinc can also regulate HIF-1α. Zinc is a vital micronutrient that plays a significant role in metabolic processes within the human body. It ranks as the second most prevalent trace element, following iron, and is predominantly located in skeletal muscle and bone (MacDonald, 2000). Zinc serves as a crucial cofactor for a variety of enzymes, thereby contributing significantly to numerous biochemical pathways. Furthermore, zinc is integral to processes such as cell proliferation, the synthesis of DNA and RNA, gene expression, the preservation of structural integrity, and the comprehensive regulation of the immune system (Li et al., 2013).

As DKD advances, there is a progressive decline in the glomerular filtration rate, accompanied by a reduction in plasma zinc levels (Nie et al., 2022). Furthermore, the diminished absorption of zinc in the intestines or the heightened urinary excretion of zinc in individuals with diabetes may contribute to a decrease in blood zinc levels among this population. A comparative analysis between patients with DKD and healthy individuals revealed that the plasma zinc ion concentration in DKD patients is significantly lower than that observed in healthy subjects (Nie et al., 2022). In patients undergoing hemodialysis, the prevalence of zinc deficiency ranges from 40% to 70%. Additionally, individuals with DKD frequently exhibit hypozincemia, particularly in developing nations. The elevated occurrence of hypozincemia may significantly contribute to the rising incidence of diabetes (Tang et al., 2010).

Numerous studies have suggested a possible interaction between zinc and diabetes, along with associated complications (Aschner et al., 2023; Nardinocchi et al., 2010). Physiological concentrations of zinc may confer advantages for individuals diagnosed with diabetes. Research, encompassing both *in vitro* and *in vivo* studies involving animal and human subjects, indicates that zinc has a variety of beneficial effects on both type 1 and type 2 diabetes (Takahashi, 2024; Yang et al., 2017). In a study, researchers administered zinc oxide nanoparticles at a dosage of 10 mg/kg/day orally to diabetic rats over a period of four consecutive weeks. Following the treatment, there was a significant enhancement in kidney function among the diabetic rats, accompanied by a notable reduction in blood glucose levels, blood urea nitrogen (BUN), serum creatinine, and urinary protein when compared to the control group (Abd El-Baset et al., 2023). In a separate investigation, researchers administered a daily dosage of 5 mg/kg of zinc chloride to mice with DKD. Histopathological examination of the renal tissue from the zinc-supplemented cohort revealed only mild congestion of the interstitial capillaries and minimal infiltration of mononuclear cells (Anton et al., 2022). Following a 6-week period of consistent supplementation with zinc carbonate in diabetic rats, a diet high in zinc has been shown to markedly decrease the concentrations of ROS and lipid peroxides in the renal tissues of these diabetic subjects (Barman et al., 2018). An investigation involving serum samples from 132 individuals diagnosed with T2DM, alongside *in vitro* experiments, revealed that zinc possesses the capacity to impede the propensity for calcification within the serum (Nakatani et al., 2020). Furthermore, zinc supplementation in diabetic patients has been shown to improve glycemic control and enhance lipid profiles (Takahashi, 2024). The significance of zinc in the prevention and mitigation of DKD progression has been thoroughly documented in numerous experimental investigations. The current management of DKD primarily aims to alleviate symptoms and postpone the decline in renal function. This is accomplished through the comprehensive regulation of various parameters, including blood pressure, blood glucose levels, and lipid profiles, in conjunction with symptomatic therapies designed to mitigate the progression of kidney disease. Frequently utilized pharmacological agents in clinical settings encompass RAS inhibitors, SGLT-2 inhibitors, glucagon-like peptide-1 (GLP-1) receptor agonists, dipeptidyl peptidase-4 (DPP-4) inhibitors, and selective endothelin (ET) α receptor antagonists. A detailed examination of their specific mechanisms and clinical limitations is presented in Table 2. Nevertheless, the intricate pathogenesis of DKD, coupled with the absence of targeted therapeutic interventions, results in suboptimal treatment outcomes, contributing to an increasing prevalence of ESRD. Considering the significant role of HIF-1α in the pathogenesis and progression of DKD, the subsequent analysis will succinctly examine the interaction mechanisms between the trace element zinc and the targets of HIF-1α. This exploration aims to offer potential targeted therapeutic strategies for clinical application.

**TABLE 2:** In the clinical treatment of DKD commonly used medications, their mechanisms of action, and an analysis of their limitations.

Medication	Representative drug	Mechanism	Potential toxicity or limitation
RAS inhibitor	ACEI/ARB	By inhibiting the production of Ang II, reducing blood pressure, reducing the excretion of proteinuria, protecting the kidney	Hyperkalemia and acute kidney injury
SGLT-2 inhibitor	Dapagliflozin Empagliflozin Tegemiflozin	Inhibiting SGLT-2 on the luminal side of proximal renal tubule cells inhibits glucose reabsorption promotes urinary glucose excretion and reduces blood glucose. Through volume-regulating mechanisms it constricts the afferent arterioles reducing intraglomerular pressure thereby exerting an effect of reducing proteinuria and protecting the kidney	There is an increased risk of urinary and genital system infections, and when used in combination with other drugs, there is an increased risk of hypoglycemia. Treatment of patients with type 1 diabetes increases the risk of diabetic ketoacidosis
GLP-1 receptor agonist	Liraglutide	Glucose-dependent stimulation of insulin synthesis and secretion, reducing glucagon release	Nodules at the site of injection
DPP-4 inhibitors	Rilatidine	Inhibiting DPP-4 activity, reducing endogenous GLP-1 inactivation, increasing GLP-1 levels, increasing glucose-dependent insulin secretion, and reducing postprandial glucagon secretion	Hypersensitivity reactions, elevated liver enzymes, upper respiratory tract infections, pancreatitis, arthralgia and other symptoms may occur
Selective ET α receptor antagonist	Atrasentan	Activation of ET receptors in the kidney leads to oxidative stress, podocyte damage, vasoconstriction, fibrosis and increased inflammation. Inhibition of ET receptors can delay the progression of DKD.	Sodium retention increases the risk of heart failure and readmission

### The role of zinc in DKD through the regulation of HIF-1α

**FIGURE 4:**
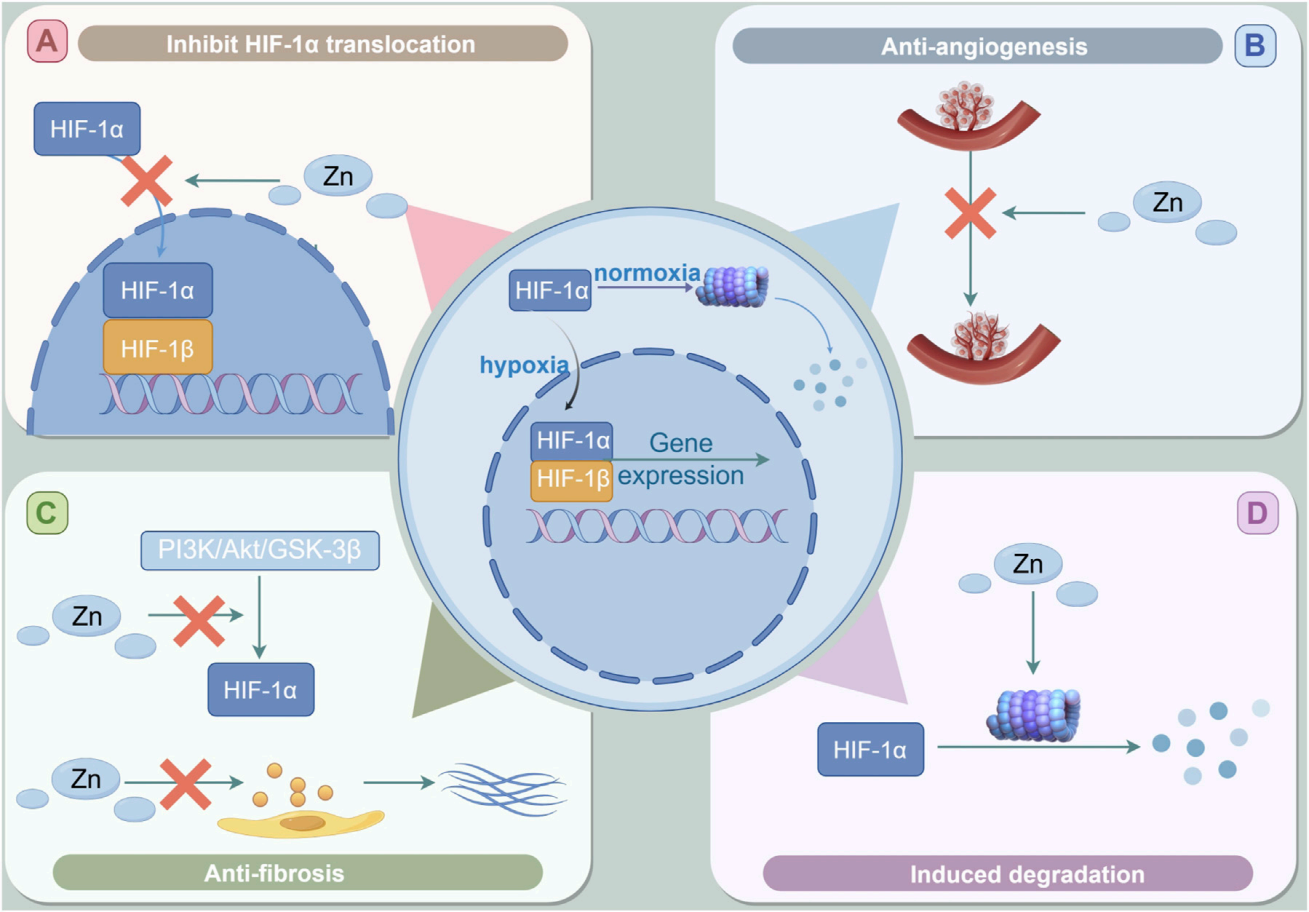
The role of Zinc in DKD through the regulation of HIF-1α. **(A)** Zinc supplementation may hinder the activation of HIF-1α by inhibiting the intranuclear shift of HIF-1α and destroying the heterodimerization of HIF-1. **(B)** Zinc can inhibit HIF-1-induced VEGF expression. **(C)** Zinc supplementation inhibits the expression of HIF-1α may involve the activation of zinc-induced PI3K/Akt/GSK-3β signal pathway.the anti-fibrosis effect of zinc may be lowered by inhibiting the activation of PI3K/Akt/GSK-3β signaling pathway to inhibit the hypoxia-induced HIF-1α accumulation and the change of EMT markers. **(D)** Zinc supplementation can reduce the expression decline of HIF-1α in cells by promoting the degradation of HIF-1α in the proteasome pathway, thus inhibiting the adverse effects of HIF-1α in the late stage of DKD.

Inhibition of HIF-1α translocation (Figure 4A).

Studies have shown that zinc inhibits the nuclear translocation of HIF-1α in astrocytes (Pan et al., 2013). In human microvascular endothelial cells, the cultivation of cells in a zinc-deficient environment, achieved through the application of the zinc-chelating agent TPEN, led to an increased intranuclear translocation of HIF-1α, heightened secretion of downstream ET, and augmented migration of endothelial cells (Morand et al., 2019). Zinc supplementation has the potential to hinder the activation of HIF-1 by obstructing the intranuclear translocation of HIF-1α and interfering with the heterodimerization of HIF-1 (Chun et al., 2001). Certain studies indicate that zinc may facilitate the accumulation and nuclear translocation of HIF-1α. However, it may concurrently inhibit the interaction of HIF-1 with DNA by impeding the nuclear translocation of HIF-1β, which subsequently leads to a decrease in the expression of the downstream target gene EPO (Guo et al., 2015).

Inhibit pathological angiogenesis (Figure 4B).

Investigations have demonstrated that the application of zinc chloride to hypoxic cancer cells results in a significant downregulation of HIF-1α protein levels and its downstream target gene, VEGF, both *in vivo* and *in vitro*. Notably, this reduction can be reversed by the proteasome inhibitor MG132. These findings indicate that zinc may facilitate the degradation of HIF-1α in hypoxic conditions via the proteasome pathway, thereby contributing to the observed decrease in HIF-1α levels (Nardinocchi et al., 2010). Researchers conducted an experiment in which primary human microvascular endothelial cells were cultured in a low zinc environment, induced by the chelator TPEN, for a duration of 5 hours. They observed a notable increase in the nuclear content of HIF-1α, as evidenced by immunofluorescence analysis. Furthermore, quantitative PCR analysis revealed that following TPEN treatment, the levels of the downstream target gene ET-1, associated with HIF-1α, were significantly elevated in the cell supernatant. This increase was found to be inhibited by the application of HIF-1α specific siRNA. These findings indicate that zinc plays a critical role in modulating the formation of the HIF-1 heterodimer and the expression of the downstream target gene ET-1 by influencing the translocation of HIF-1α into the nucleus of the cells (Morand et al., 2019). Homeodomain-interacting protein kinase 2 (HIPK2) has been identified as a potential biomarker for tumor proliferation. The research indicates that HIPK2 is capable of interacting with the promoter region of HIF-1, thereby inhibiting its activation. This interaction results in the downregulation of transcription of the downstream target genes of HIF-1 (Nardinocchi et al., 2009). Zinc supplementation has been shown to counteract the transcriptional repression of HIPK2 induced by hypoxic conditions in cancer cells, leading to a decrease in the expression of HIF-1α (Hatano et al., 2021).

Previous research has indicated that elevated levels of HIF-1α can enhance the expression of the downstream target gene VEGF, potentially resulting in pathological angiogenesis and subsequent renal fibrosis. Empirical studies have demonstrated that zinc supplementation can mitigate pathological angiogenesis and ameliorate renal fibrosis through the inhibition of the HIF-1α/VEGF signaling pathway.

Anti-fibrosis (Figure 4C).

A substantial body of research has indicated that zinc supplementation may effectively mitigate fibrosis across multiple tissue types (Malekahmadi et al., 2024). In previous investigations, we administered zinc supplementation and implemented zinc deficiency interventions in animal models of DKD, specifically in rats and mice, as well as in cellular models characterized by high glucose and high fat conditions. These studies corroborated the efficacy of zinc in mitigating fibrosis within diabetic renal tissues (Li et al., 2014; Wang et al., 2020). This aligns with findings from another study, which demonstrated that a 3-month regimen of zinc supplementation in diabetic rats led to a reduction in histological alterations, a significant decrease in the expression of fibrotic markers such as connective tissue growth factor, and an upregulation of metal-binding proteins in both cardiac and renal tissues (Tang et al., 2010).

The preceding discussion has elucidated the role of HIF-1α in facilitating the advancement of renal fibrosis through multiple mechanisms. Research indicates that zinc supplementation can mitigate renal tubular EMT and renal tubular interstitial fibrosis by downregulating HIF-1α in models of DKD (Zhang X et al., 2016). Specifically, Zhang et al. demonstrated that zinc supplementation in DKD rat models and in high glucose/hypoxia-induced cellular models significantly reduced the expression of fibrotic markers, including fibronectin, α-SMA, and E-cadherin. Han et al. found that both HIF-1α siRNA and the overexpression of PHD2 can effectively block the loss of epithelial markers and the increase in the expression of mesenchymal markers induced by TGF-β1 in renal tubular cells cultured *in vitro* (Han et al., 2013). In the cellular model, the antifibrotic effects of zinc were negated following treatment with PI3K inhibitors such as LY 294002 or wortmannin, which resulted in the restoration of E-cadherin levels and a reduction in α-SMA expression. This observation implies that zinc supplementation may downregulate HIF-1α expression and inhibit the activation of the PI3K/Akt/glycogen synthase kinase-3β (GSK-3β) signaling pathway, thereby diminishing the hypoxia-induced accumulation of HIF-1α and alterations in EMT markers, ultimately suppressing EMT in cells subjected to high glucose and hypoxia. The proposed mechanism of action may involve the mediation of zinc’s negative regulatory effects on the HIF-1α signaling pathway by disrupting the critical function of the zinc finger domain in PHD. Conversely, zinc deficiency, induced by the zinc chelator TPEN in primary human microvascular endothelial cells, has been shown to upregulate HIF-1α signaling, potentially through enhanced nuclear translocation of HIF-1α and increased secretion of ET-1 (Guo et al., 2015).

Zinc-induced proteasome degradation (Figure 4D).

In a standard oxygen environment, HIF-1α undergoes hydroxylation by PHD enzymes, which subsequently leads to its degradation via the proteasome, facilitated by the action of the VHL ubiquitin ligase. As a result, the detection of HIF-1α under normoxic conditions presents significant challenges. Some researchers have employed the proteasome inhibitor MG132 in cellular treatments, revealing that the impact of zinc supplementation on reducing HIF-1α levels can be negated by the presence of proteasome inhibitors. This finding suggests that zinc may modulate HIF-1α levels through the proteasomal degradation pathway (Nardinocchi et al., 2010). In a separate investigation, it was found that zinc supplementation inhibits the interaction between HIF-1α and the VEGF promoter by promoting the degradation of HIF-1α via the proteasome pathway. Furthermore, the introduction of a zinc chelating agent resulted in an increase in HIF-1α expression within cells, an effect that could be reversed by the addition of zinc. This indicates that zinc supplementation may mitigate the decline in HIF-1α expression in cells by enhancing its degradation through the proteasome pathway, thereby alleviating the adverse effects associated with HIF-1α during the later stages of DKD (Aschner et al., 2023; Nardinocchi et al., 2010).

### The potential toxicity of zinc

The consumption of zinc is not without its drawbacks, as potential adverse effects may restrict its application. A study conducted in China identified a U-shaped correlation between dietary zinc intake and the risk of diabetes, with a critical threshold established at 9.1 mg/d (He et al., 2022). Excessive zinc consumption has been associated with an increased risk of developing diabetes. Furthermore, research indicates that prolonged hyperzincemia may elevate the risk of thrombosis (Hughes and Samman, 2006). An experimental study involving 11 healthy adult males demonstrated that continuous administration of 300 mg of zinc sulfate could result in reversible impairments in immune function (Chandra, 1984). *In vitro* investigations have revealed that elevated intracellular zinc levels can directly induce apoptosis in endothelial cells (Wiseman et al., 2010).

Common side effects associated with zinc supplementation include nausea, vomiting, and pruritus, with the symptoms of nausea and vomiting being mitigated when zinc is ingested postprandially. Additionally, increased zinc concentrations may lead to reduced serum copper levels (Koi et al., 2020), potentially resulting in copper deficiency-related anemia and leukopenia (Gembillo et al., 2022; Kodama et al., 2020). The proposed mechanism for this phenomenon suggests that excessive zinc intake enhances the binding of zinc to metallothionein in intestinal epithelial cells, which subsequently interferes with copper binding to metallothionein, thereby inducing copper deficiency (Duncan et al., 2015). Abnormalities in copper metabolism may compromise the activity of zinc-copper superoxide dismutase, consequently impairing antioxidant functions. Future research must delve deeper into the optimal intake levels of zinc and the potential mechanisms linking zinc to DKD.

## Conclusion

DKD represents a significant complication associated with diabetes mellitus. The prevailing standard of care primarily emphasizes the management of glycemic levels, blood pressure, and lipid profiles. Nevertheless, a considerable number of patients continue to advance to ESRD, even while undergoing these interventions. Consequently, comprehensive investigations into the pathophysiology of DKD and the identification of potential therapeutic targets are crucial for the effective prevention and management of this condition.

The incidence of DKD is intricately linked to hemodynamic disturbances, metabolic irregularities, and the consequent inflammatory responses and oxidative stress, which collectively contribute to a hypoxic milieu within the renal system. HIF-1, a pivotal regulator of hypoxic responses, plays a significant role in angiogenesis, erythropoiesis, oxidative stress, inflammatory processes, mitochondrial autophagy, and extensive fibrosis by inducing aberrant expression of its downstream target genes, including VEGF, EPO, and HO-1. This involvement underscores the potential of HIF-1α as a therapeutic target for DKD. Currently, pharmacological agents aimed at HIF-1α are being utilized in the management of this condition. Roxadustat, a drug classified as a HIF-PHI, has progressed to clinical research phases, primarily for the treatment of renal anemia associated with CKD. HIF-1α mediates damage in DKD through various extensive signaling pathways, necessitating further investigation into targeted therapies for these alternative pathways. Although research on HIF-1α and DKD has gradually increased in recent years, many enigmas remain unresolved. For instance, does HIF-1α exert non-transcriptional effects while mediating hypoxic adaptation in a high-glucose and hypoxia environment? How can we effectively visualize the levels of PO_2_ within renal cells? Furthermore, the mechanisms, translational potential, and clinical exploration of treating DKD through the modulation of HIF-1α activity still warrant deeper investigation.

Patients suffering from DKD frequently exhibit dysregulated zinc metabolism. Existing literature suggests that zinc supplementation may exert beneficial effects on DKD. Zinc influences the expression of HIF-1α in cells subjected to hyperglycemic and hypoxic conditions through multiple mechanisms, including the inhibition of HIF-1α translocation, as well as exhibiting anti-inflammatory, antioxidant, anti-apoptotic, and anti-fibrotic properties. Consequently, zinc may represent a viable therapeutic strategy for modulating HIF-1α and decelerating the progression of DKD. However, current investigations into the efficacy of zinc predominantly focus on animal and cellular models, highlighting the necessity for further clinical research in human populations to elucidate the specific mechanisms by which zinc supplementation may affect DKD. While some studies have indicated a potential renal protective effect of zinc supplementation in DKD patients, there remains a pressing need for more randomized controlled trials with larger cohorts and extended follow-up durations to ascertain the long-term effects of zinc treatment. Given that zinc supplementation may negatively impact copper metabolism, additional clinical research is urgently required to establish the optimal zinc intake levels for DKD patients to maintain a balance between copper and zinc. Furthermore, under pathological conditions, the pharmacokinetics and pharmacodynamics of zinc supplementation may be altered by changes in the internal environment, necessitating comprehensive animal studies and clinical trials to identify the most effective zinc supplementation concentrations for patients with varying severities of DKD.

Zinc supplementation represents a promising area for further investigation. Currently, commonly utilized zinc supplements in clinical practice include zinc sulfate, zinc chloride, zinc nitrate, zinc gluconate, zinc acetate, and zinc lactate. Additionally, zinc oxide nanoparticles present a novel and effective treatment strategy for DKD, owing to their ability to address the limitations associated with the short duration of action of oral medications, low oral bioavailability, and significant side effects. However, current research on the application of zinc oxide nanoparticles in the treatment of DKD is relatively scarce, and the safety of this treatment process, along with associated adverse reactions, must be further validated through extensive animal experiments and clinical studies.

## Glossary

α-SMA: α-smooth muscle actin
ACEI: angiotensin-converting enzyme inhibitor
AGEs: advanced glycation end products
AngⅡ: angiotensin II
ARB: angiotensin II receptor blocker
ATP: adenosine triphosphate
Bcl-2: B-cell lymphoma-2
Bcl-xL: B-cell lymphoma-extra large
BNIP3: Bcl-2 interacting protein 3
BOLD-MRI: blood oxygenation level-dependent magnetic resonance imaging
BUN: blood urea nitrogen
CKD: chronic kidney disease
DKD: diabetic kidney disease
DPP-4: dipeptidyl peptidase-4
ECM: extracellular matrix
EMT: epithelial-mesenchymal transition
EPO: erythropoietin
ERS: endoplasmic reticulum stress
ESRD: end-stage renal disease
ET: endothelin
FIH-1: factor-inhibiting HIF-1
GLP-1: glucagon-like peptide-1
GSK-3β: glycogen synthase kinase-3β
HIF-1: hypoxia-inducible factor-1
HIF-PHI: HIF-PHD inhibitors
HIPK2: homeodomain-interacting protein kinase 2
HO-1: heme oxygenase-1
HRE: hypoxia response element
IL-1: interleukins 1
IL-1β: interleukin-1β
IL-6: interleukin-6
IL-18: interleukin-18
KLF5: Krüppel-like factor 5
LOXL-2: lysyl oxidase-like 2
MCP-1: monocyte chemoattractant protein-1
NF-κB: nuclear factor-κB
NLRP3: nucleotide-binding oligomerization domain-like receptor protein 3
PAI: plasminogen activator inhibitor
PERK: protein kinase R-like endoplasmic reticulum kinase
PHD: prolyl hydroxylase
PI3K/AKT: phosphatidylinositol 3-kinase/protein kinase B
RIF: renal interstitial fibrosis
ROS: reactive oxygen species
RUNX2: runt-related transcription factor 2
SGLT: sodium-glucose co-transporter
SOD: superoxide dismutase
TGF-β1: transforming growth factor-β1
TIMP: tissue inhibitors of metalloproteinase
TNF-α: tumor necrosis factor-α
TXNIP: thioredoxin-interactingprotein
T2DM: type 2 diabetes mellitus
VEGF: vascular endothelial growth factor
VHL: von Hippel-Lindau
VSMCs: vascular smooth muscle cells
